# HIV Interacts with Posttraumatic Stress Disorder to Impact Fear Psychophysiology in Trauma-Exposed Black Women

**DOI:** 10.1089/whr.2023.0133

**Published:** 2024-03-12

**Authors:** Susie Turkson, Sanne J.H. van Rooij, Abigail Powers, Ighovwerha Ofotokun, Seth D. Norrholm, Gretchen N. Neigh, Tanja Jovanovic, Vasiliki Michopoulos

**Affiliations:** ^1^ Department of Anatomy and Neurobiology, Virginia Commonwealth University, Richmond, Virginia, USA.; ^2^ Department of Psychiatry and Behavioral Sciences, Emory University School of Medicine, Atlanta, Georgia, USA.; ^3^ Division of Infectious Diseases, Department of Medicine, Emory University School of Medicine, Atlanta, Georgia, USA.; ^4^ Grady Health System, Atlanta, Georgia, USA.; ^5^ Department of Psychiatry and Behavioral Neurosciences, Wayne State University, Detroit, Michigan, USA.; ^6^ Emory National Primate Research Center, Atlanta, Georgia, USA.

**Keywords:** HIV, childhood trauma, women, PTSD

## Abstract

**Background:**

The prevalence of posttraumatic stress disorder (PTSD) among people living with HIV (PLWH) is higher than in the general population and can impact health behaviors. The influence of HIV on PTSD psychophysiology requires further investigation due to implications for the treatment of PTSD in PLWH.

**Objective:**

Utilizing fear-potentiated startle (FPS), we aimed to interrogate the influence of PTSD and HIV on fear responses.

**Materials and Methods:**

Women (18–65 years of age) recruited from the Women's Interagency HIV Study in Atlanta, GA (*n* = 70, 26 without HIV and 44 with HIV), provided informed consent and completed a semistructured interview to assess trauma exposure and PTSD symptom severity. Participants also underwent an FPS paradigm to assess fear acquisition and extinction: Psychophysiological indices that measure how individuals learn new fear and then subsequently attempt to suppress this fear.

**Results:**

Women with PTSD, who did not have HIV, exhibited a greater startle response compared to women without PTSD or HIV during late acquisition to both the danger cue, reinforced conditioned stimulus (CS+, *p* = 0.013)), and the safety cue, non-reinforced conditioned stimulus (CS–, *p* = 0.046)), whereas women living with HIV (WLH) and PTSD demonstrated blunted fear responses compared to women with PTSD only. During extinction, WLH comorbid with PTSD exhibited an increased fear response during the extinction period in comparison to all other groups (*p* = 0.023). Women without PTSD demonstrated a reduction in the fear response during extinction regardless of HIV status.

**Conclusion:**

Our findings indicate that HIV further modifies fear psychophysiology in WLH with comorbid PTSD, highlighting the importance of considering HIV status in conjunction with PTSD treatment.

## Introduction

Although the worldwide numbers of newly diagnosed HIV infections continue to grow,^1^ the effectiveness and expansion of antiretroviral therapy (ART) for HIV have made HIV more manageable, and ∼39 million people currently live with HIV worldwide. While ART has reduced AIDS-related morbidity and mortality, HIV has become a lifelong condition that can impact other aspects of overall health. One factor that decreases adherence to ART and increases odds of virologic failure is exposure to stressful life events,^2^ including trauma. Close to 90% of people living with HIV (PLWH) experience significant trauma during their lifetime,^3^ a risk factor for the development of posttraumatic stress disorder (PTSD).^4,5^ Both trauma exposure and PTSD development are associated with ART nonadherence in PLWH.^6–9^ Furthermore, PTSD increases the risk of other chronic health conditions, such as cardiometabolic disorders, which are also highly prevalent in PLWH.^6–9^

PTSD is a heterogeneous psychiatric condition that is characterized by a combination of intrusive and fearful memories, flashbacks, and nightmares of the traumatic event(s), as well as avoidance and numbing, hyperarousal symptoms, and negative cognitions and mood.^4^ Although initially identified among combat veterans, research now indicates that several populations are at high risk for PTSD based on high levels of trauma exposure, with highest rates among underresourced and minoritized Black women living in urban environments.^10,11^ Unfortunately, minoritized Black women living in urban environments are also at an increased risk of HIV infection,^12,13^ and Black women make up roughly 55% of women living with HIV (WLH) in the United States.^13^ In addition, many WLH report having experienced trauma and roughly 30% have been diagnosed with PTSD.^9^

The etiology and maintenance of PTSD are linked to psychophysiological hyperarousal in the presence of cues or contexts that have been associated with a psychological trauma, such that one's evaluation of threat and physiological reactivity can become significantly elevated.^14^ Elevated psychophysiological responses to emotionally salient stimuli have been robustly examined in our previous work with traumatized populations and this work has revealed fear-potentiated startle (FPS) to be a putative intermediate phenotype for posttraumatic stress reactions, including PTSD.^15^ The acoustic startle response is an inborn reflexive contraction of the skeletal musculature following exposure to a sudden loud auditory stimulus such as a burst of white noise,^16^ a defensive reaction present in all mammals.

In humans, this is typically captured by measuring the blink response of the eyes in the presence of a loud noise. FPS refers to an increase in the magnitude of the acoustic startle response (*e.g.*, stronger blinks) in the presence of cues or contexts that have been associated with increased threat, fear, or anxiety.^15^ For example, FPS is routinely seen in the presence of stimuli (*e.g.*, geometric shapes presented on a computer screen) that have been repeatedly paired with an aversive outcome (*e.g.*, electric shock applied to the skin or puff of air directed at the throat) through classical conditioning.^25,26^ In this type of classical conditioning paradigm, the geometric shapes are termed conditioned stimuli (CSs) and the aversive outcomes are referred to as unconditioned stimuli (USs).

The learning of new fear to a CS (shape) that has been repeatedly paired with a US (airblast) is called fear acquisition and is evidenced as an increased startle in the presence of the paired CS (called the CS+). Specificity of the fear response to a particular CS is confirmed with the presentation of a second shape that is not paired with an aversive outcome (termed the CS–).

Colloquially speaking, the CS+ is considered a danger signal, whereas the CS– is conceptualized as a safety signal.^17^ Startle responses are derived under baseline conditions (no shapes) and upon individual presentations of the danger or safety signals (*e.g.*, CS+ or CS–), such that any change in startle from baseline in the presence of a CS indicates an increased or decreased level of fear. In psychiatrically healthy controls, startle will be potentiated under conditions of danger (*e.g.*, CS+ presentations) compared to baseline and startle to danger (*e.g.*, CS+) will be greater than startle to safety (*e.g*., CS– presentations).^18^

Following successful acquisition of new fear to the CS+, investigators can then assess one's ability to suppress or extinguish this new fear through the repeated presentation of the CS+ or danger signal without any aversive outcome. In short, participants learn what was once “dangerous” can now be considered “safe”’ within the laboratory procedures. This process is called fear extinction and is the learning principle that underlies clinical exposure therapies for fear and anxiety.^18–20^ Our group and others have previously shown fear acquisition and extinction learning to be impaired in traumatized populations with PTSD (for review, see Ref.^21^). More specifically, in traumatized Black women with PTSD, we have seen exaggerated startle to the danger signal (CS+) during the later stages of fear acquisition and during fear extinction through a phenomenon that we have termed “fear load.”^14^

While greater FPS during late acquisition and deficits in fear extinction have been well established in PTSD, the potential modulating role of HIV status on fear psychophysiology has not been investigated. The lack of knowledge surrounding how HIV status influences fear regulation in individuals with PTSD may limit the generalizability of treatment strategies for WLH and PTSD.

Understanding the extent to which HIV infection modulates fear acquisition and extinction will better inform providers with critical information with which to best approach PTSD treatment for WLH. Given that Black women are historically understudied in both the context of HIV and PTSD and at risk for both conditions,^22,23^ we focused specifically on this population. The goal of this study was to determine whether HIV interacts with PTSD to influence psychophysiological fear responses, as assessed by FPS in trauma-exposed Black women with and without HIV. We hypothesized that WLH and PTSD would show exaggerated fear acquisition and the greatest deficits in fear extinction.

## Materials and Methods

### Participants

Seventy Black women (26 without HIV and 44 with HIV [WLH]) between 18 and 65 years of age were recruited from the Women's Interagency HIV Study in Atlanta, GA, USA. All participants provided informed consent. Medical data regarding HIV status and viral load (quantified by polymerase chain reaction) at the time of enrollment in this study were collected. Exclusion criteria included the following: Active symptoms of mania, schizophrenia, or other psychoses; current prominent suicidal ideation; intoxication; special medical conditions that can contribute significantly to psychiatric symptoms, including hypothyroidism or hyperthyroidism, systemic lupus erythematosus, cirrhosis, and dementia. We also excluded participants with positive urine drug screen, loss of consciousness for more than 5 minutes, and failed hearing screen for the startle testing. All study procedures were approved by the Emory Institutional Review Board and the Grady Hospital Research Oversight Committee.

### Clinical assessment measures

All participants completed a clinical assessment conducted by a staff trained to administer all psychological assessment instruments. Demographic information was collected using a locally developed demographics form to assess self-reported age, race, education, and income.^24^ The clinical assessment included the Traumatic Events Inventory (TEI) and the PTSD Checklist (PCL-5). The 14-item TEI was administered to assess lifetime history of trauma exposure.^24^

The TEI is a validated measure of trauma exposure with construct validity, as higher levels of reported trauma using this measure is associated with more severe symptoms of relevant constructs such as PTSD symptoms.^25^ For this study, total types of trauma exposure experienced and witnessed were summed across different types of trauma (including exposure to a life-threatening illness, such as HIV) to measure lifetime trauma exposure. Higher values in the summed TEI score indicate greater exposure to different types of traumas over the life course. The PCL-5 for DSM-5 (Diagnostic and Statistical Manual of Mental Disorders, 5th Edition)^26,27^ is a 20-item self-report questionnaire based on the DSM-5 symptom criteria for PTSD and has been validated in a minoritized sample.^28^ The PCL-5 was used to determine the presence of a provisional PTSD diagnosis.^26,27^

### FPS paradigm

The FPS paradigm was used to assess psychophysiological hyperarousal and deficits in physiological learning. FPS was measured by the relative increase in the acoustic startle reflex in the presence of a CS, which has been paired with an aversive US.^17^ Differential fear conditioning was conducted by using two distinct cues: The reinforced conditioned stimulus (CS+, also referred to as the danger signal) and a non-reinforced conditioned stimulus (CS–, also referred to as the safety signal). Participants were individually seated in a sound attenuated booth with a computer monitor ∼1 m in front of them and they were asked to look at the monitor. The startle probe was a 40-ms burst of broadband white noise at 108 dB, delivered binaurally through headphones.

#### Startle testing

Electromyographic (EMG) data were sampled at 1000 Hz and amplified using the Biopac MP150 system (Biopac Systems, Inc., Aero Camino, CA, USA). The acquired data were filtered and rectified in MindWare software (MindWare Technologies, Inc.) and exported for statistical analyses. EMG activity was recorded from two 5 mm Ag/AgCl electrodes placed over the orbicularis oculi muscle, ∼1 cm under the pupil and 1 cm below the lateral canthus. The impedances for all participants were <6 kΩ. The EMG signal was filtered with cutoffs at 28 and 500 Hz, for low and high frequency, respectively. Our measure of the acoustic startle response was the startle magnitude assessed as the peak amplitude of the EMG contraction 20–200 ms, following the acoustic stimulus, as previously described.^17^

#### Fear acquisition

**FIG. 1.**
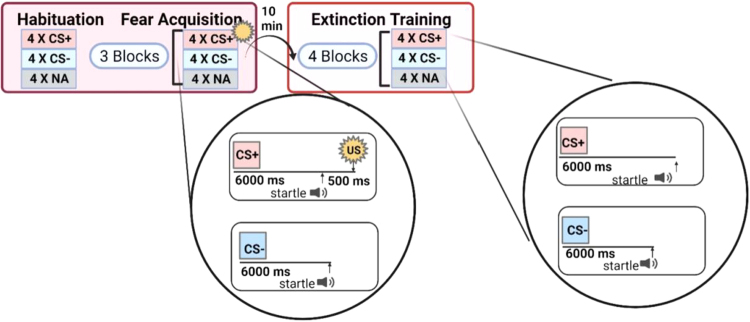
Diagram of fear-potentiated startle paradigm **(**created in BioRender.com).

After an initial habituation phase wherein CS was presented without any reinforcement,^14,29^ fear acquisition was initiated. The fear acquisition phase consisted of three blocks with four trials of each type of CS (CS+; CS–; noise probe alone [NA]) for 12 trials per block and a total of 36 trials (Fig. 1). Block 3 of acquisition is defined as late acquisition, when discrimination learning was at a maximum. Both CSs were colored shapes (*i.e.*, blue square, purple triangle) presented on a computer monitor for 6 seconds each before the delivery of the startle probe and counterbalanced across subjects.

The US (aversive stimulus) was a 250-ms air blast of 140-psi intensity to the larynx delivered from a compressed air tank by polyethylene tubing, controlled by a solenoid switch. The US was paired with the CS+ on 100% of acquisition trials and has previously been shown by our group to elicit a strong FPS response.^14,29^ The FPS was designated as the change in the magnitude of the eyeblink response to the acoustic probe in the presence of a CS relative to the blink response to the acoustic probe in the absence of a conditioned stimulus. Specifically, the difference score, previously defined^30^ as ([startle magnitude in the presence of a CS in each conditioning block] – [startle magnitude to the NA]), was used to calculate FPS.

#### Fear extinction

Ten minutes after the fear acquisition session, the fear extinction session occurred.^14,29^ The fear extinction phase included the same CS+ and CS– trials, except that the CS+ was no longer paired with the US. The extinction phase was composed of 48 trials, with four blocks of 12 trials of each type (NA, CS+ [unreinforced], and CS–) in each block (Fig. 1). Percent extinction was calculated as (block 1 – block 4)/block 1) × 100, to test group differences in the degree of fear reduction normalized by the level of fear at the start of extinction.

### Data analysis

Data are summarized as mean ± standard error of the mean. SEM. Sociodemographic data were compared for HIV serostatus using *t*-tests and chi-square tests and variables that were significantly different between those with and without HIV were used as covariates. A repeated-measures analysis of variance test (ANOVA) using the within-subject factor of block (three levels, 1 vs. 2 vs. 3) and trial type (two cues: CS+ and CS-) was used to analyze the effects of block and trial type on FPS during fear acquisition. Repeated-measures analyses of covariance (ANCOVA), covarying for income and education (found to be different between those with and without HIV; see section 3.1 below), were used to assess the impact of HIV, PTSD, and their interaction on late fear acquisition of CS+ and CS-, separately.

For fear extinction, a repeated-measures ANOVA to assess the impact of block (four levels: 1 vs. 2 vs. 3 vs. 4) and trial type (two levels: CS+ and CS–) was conducted. An ANCOVA adjusting for income and education was run to assess the impacts of HIV, PTSD, and their interaction on percent fear extinction. Effect sizes for the analyses are shown as partial eta squared (η^2^). Bivariate correlations between PTSD symptom total scores and percent extinction were run and stratified by HIV status to analyze the relationships between PTSD symptom score and percent fear extinction. All statistical analyses were performed in SPSS 29.0 for Windows (SPSS, Inc., Chicago, IL, USA) and α-level was set to *p* < 0.05 for statistical significance.

## Results

### Sociodemographic characteristics

Of the 70 women enrolled, there were 44 WLH (35 women had HIV only and 9 had both HIV and PTSD) and 26 women without HIV (4 had PTSD only, and 22 women had neither PTSD nor HIV). There was no significant difference in age based on HIV, PTSD, or their interaction (*p'*s > 0.05; Table 1). Rates of lifetime trauma exposure and probable PTSD diagnosis were not significantly different based on HIV, PTSD, or their interaction (*p'*s > 0.05; Table 1).

**Table 1.** Demographic Characteristics for Sample by HIV Serostatus and Probable Posttraumatic Stress Disorder

	HIV−				HIV+				
	PTSD−		PTSD+		PTSD−		PTSD+		
Factor	Mean ± SEM	*N*	Mean ± SEM	*N*	Mean ± SEM	*N*	Mean ± SEM	*N*	*p*-value
Age	48 ± 2	22	41 ± 3	4	49 ± 2	35	52 ± 2	9	0.09
Lifetime Trauma exposure (TEI)	4.91 ± 0.75	22	7.25 ± 2.87	4	5.43 ± 0.69	35	4.80 ± 0.94	9	0.25
PTSD symptoms	7 ± 1	22	32 ± 3	4	8 ± 1	35	29 ± 2	9	0.26
	%	*N*	%	*N*	%	*N*	%	*N*	
Virally suppressed					91.4	35	88.9	9	0.81
	%			*N*					
Probable PTSD	15.4			4	20.5			9	0.59
	%	*N*	%	*N*	%	*N*	%	*N*	
Education									0.55
Less than high school degree	40.9	9	25	1	20	7	0	0	0.39
Graduated high school or GED	31.8	7	50	2	17.1	6	62.5	5	0.28
Some college, college degree, or graduate degree	27.3	6	25	1	62.9	22	37.5	3	0.87
Income ($/month)									0.01^a^
<$500	27.3	6	25	1	5.7	2	25	2	0.20
$500–$1000	22.7	5	25	1	34.3	12	37.5	3	0.86
>$1000	50	11	50	2	60	21	37.5	3	0.81

*p*-values reported for the interaction term.

^a^ *p* < 0.05.

PTSD, posttraumatic stress disorder; TEI, Traumatic Events Inventory.

There was a main effect of PTSD on total PTSD symptoms, such that a higher total of PTSD symptoms was reported for individuals who met criteria for probable PTSD (*p* < 0.001), but no significant difference based on HIV or the interaction of HIV and PTSD (*p'*s > 0.05; Table 1). However, WLH had higher levels of income (*p* = 0.048) and education (*p* = 0.003) compared to women without HIV. There was no main effect of PTSD and no interaction of HIV and PTSD on income and education levels (*p'*s > 0.05; Table 1).

### Fear acquisition

**FIG. 2.**
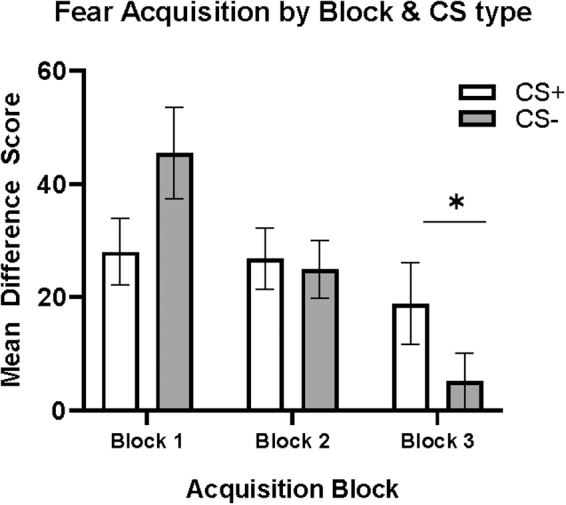
Fear acquisition. Block by type interaction and main effect of CS type in block 3, with higher FPS response to CS+ compared to CS– (*p* = 0.002; denoted by *). CS, conditioned stimulus; FPS, fear-potentiated startle.

Fear acquisition data were obtained for all 70 participants. Participants demonstrated learning of the CS+ and CS– across the blocks of acquisition, as evidenced by a significant block by CS-type interaction (F_2,69_ = 9.054, *p* < 0.001, η^2^ = 0.116), such that FPS to CS+ compared to CS– was higher during the last block of acquisition (in late acquisition; F_1,69_ = 9.958, *p* = 0.002, η^2^ = 0.126; Fig. 2). Group analyses were conducted to examine the impacts of HIV, PTSD, and their interaction on late acquisition of CS+ and CS– separately.

**FIG. 3.**
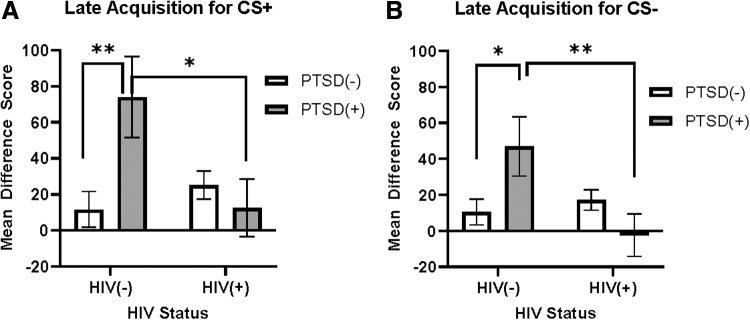
Interaction effects of HIV status and PTSD on late fear acquisition. **(A)** HIV by PTSD on CS+ FPS interaction (*p* = 0.016), such that women without HIV, but with PTSD, exhibited a greater startle response to the CS+ compared to women without HIV or PTSD (*p* = 0.013; denoted by **) and WLH and PTSD (*p* = 0.030; denoted by *). **(B)** HIV by PTSD interaction on CS–FPS interaction (*p* = 0.014), such that women without HIV, but with PTSD, exhibited a greater startle response to the CS– compared to women without HIV or PTSD (*p* = 0.046; denoted by *) and WLH and PTSD (*p* = 0.018; denoted by **). PTSD, posttraumatic stress disorder.

The ANCOVA covarying for income and education for the CS+ resulted in a significant interaction between HIV and PTSD status (F_1,63_ = 6.11, *p* = 0.016, η^2^ = 0.088), such that women with probable PTSD diagnosis, but without HIV, exhibited a greater startle response to the CS+ during the late acquisition phase compared to women without HIV or probable PTSD (F_1,63_ = 6.509, *p* = 0.013, η^2^ = 0.094; Fig. 3A) and WLH and probable PTSD (F_1,63_ = 4.93, *p* = 0.030, η^2^ = 0.094; Fig. 3A). Similarly, for CS–, a significant interaction between HIV and PTSD was observed (F_1,63_ = 6.387, *p* = 0.014, η^2^ = 0.092).

Women with probable PTSD, but without HIV, exhibited a greater startle response to the CS– during the late acquisition phase compared to both women without HIV or probable PTSD, that is, nondiagnostic controls (F_1,63_ = 4.135, *p* = 0.046, η^2^ = 0.062; Fig. 3B) and the comorbid PTSD and HIV women (F_1,63_ = 5.930, *p* = 0.018, η^2^ = 0.086; Fig. 3B).

### Fear extinction

Complete fear extinction data were not obtained from two participants, dropping the sample size to 68 (43 WLH [34 with HIV only and 9 with HIV and probable PTSD] and 25 women without HIV [4 with probable PTSD and 21 with neither HIV nor PTSD]). A trend toward main effect of CS type was observed (F_1,67_ = 3.769, *p* = 0.056, η^2^ = 0.053), demonstrating discrimination between CS+ and CS– during extinction. Group analyses were conducted to examine the impacts of HIV, PTSD, and their interaction on fear extinction.

**FIG. 4.**
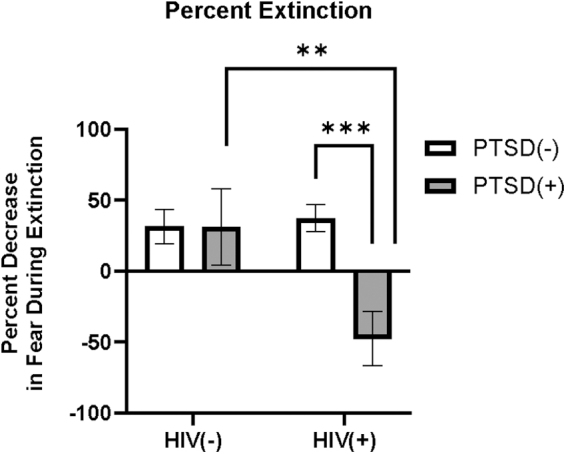
Effects of HIV status and PTSD on percent fear extinction. Interaction of HIV and PTSD on percent decrease in fear during extinction (*p* = 0.023), such that WLH and PTSD exhibited impaired fear extinction in comparison to WLH without PTSD (*p* < 0.001; denoted by***) and women with PTSD, without HIV (*p* = 0.020; denoted by **). WLH, women living with HIV.

The ANCOVA covarying for income and education for percent decrease in fear during extinction resulted in a significant interaction between HIV and PTSD status (F_1,58_ = 5.413, *p* = 0.023, η^2^ = 0.082), such that WLH and probable PTSD exhibited a greater deficit in percent fear extinction PTSD in comparison to WLH without probable PTSD (F_1,61_ = 15.8, *p* < 0.001, η^2^ = 0.206; Fig. 4) and women with probable PTSD living without HIV (F_1,61_ = 5.671, *p* = 0.020, η^2^ = 0.085; Fig. 4).

**FIG. 5.**
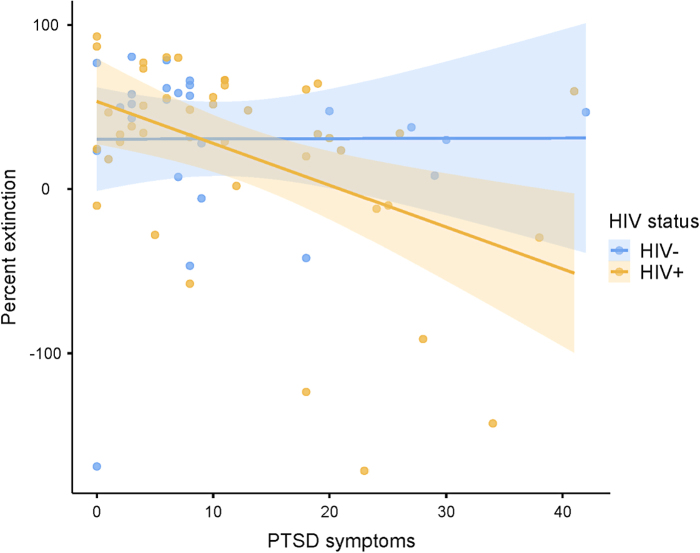
Correlation plot of PTSD symptom total score and percent extinction, stratified by HIV status. PTSD symptom total score moderately negatively correlated with percent extinction in WLH (*r* = −0.452, *p* = 0.002), such that a higher PTSD symptom total score is correlated with a more negative percent extinction.

In addition, a main effect was observed for PTSD status (F_1,66_ = 5.568; *p* = 0.022; η^2^ = 0.084), such that lower fear extinction was observed in women with probable PTSD in comparison to women without probable PTSD. Bivariate correlations between PTSD symptom total score and percent extinction revealed a moderate negative correlation between PTSD symptom total score and percent extinction in WLH (*r* = −0.452, *p* = 0.002; Fig. 5) and no correlation between the two variables in women living without HIV (*r* = 0.003, *p* = 0.998; Fig. 5).

## Discussion

This cross-sectional study highlights the interaction of HIV and PTSD on fear psychophysiology in trauma-exposed Black women. Among women without HIV infection, those living with probable PTSD demonstrated exaggerated fear acquisition. In contrast, WLH and comorbid PTSD did not demonstrate differences in acquisition in comparison to the nondiagnostic controls. Overall, our findings indicate that HIV modifies the impact of PTSD on fear psychophysiology and highlight the importance of considering HIV serostatus when deciding on therapeutic treatment options for individuals with PTSD.

Consistent with previous reports, we observed a greater fear response during acquisition in women with probable PTSD such that there is a heightened fear response to both CS+ and CS– during acquisition, despite adequate discrimination between the cues.^30,31^ Fear learning is a critical survival mechanism, especially in the context of evolution as it alerts organisms to threats.^32^ While the innate purpose of fear remains unchanged, the stimuli and context that generate fear in individuals have evolved and sometimes represent circumstances that appear benign, and in individuals with PTSD, an exaggerated fear response is an indication of an overactive fear system. Contrary to our expectations, the WLH and comorbid probable PTSD demonstrated fear responses during acquisition, which were comparable to individuals without probable PTSD. This suggests that HIV comorbid with PTSD attenuates the PTSD-associated effects on fear acquisition, which has previously been reported for fear learning.^30,31^

The observation of unimpaired learning for WLH also contrasts with previous reports showing deficits in other domains of cognition in WLH even with adequate viral suppression.^33^ Interestingly, the role of the amygdala is more pronounced in fear learning than in other domains of learning, and PLWH and a history of early life stress have been reported to have enlarged amygdala volumes.^34^ Due to the small sample size of women living with probable PTSD, in this study, we could not further segment our analyses by metrics of early life stress, and it will be important to consider this variable in future studies.

In addition, we observed deficits in fear extinction in WLH with comorbid probable PTSD compared to the other groups. Generally, a decrease in FPS occurs during extinction training^35,36^; however, WLH and comorbid PTSD exhibited an increase in fear during the extinction phase. Women with probable PTSD, but without HIV, did not show fear extinction deficits and were comparable to the groups without PTSD. Previous work implicates impairments in fear inhibition as a psychophysiological biomarker for PTSD susceptibility,^37^ and numerous studies have shown this impairment in PTSD^29,37–39^; in contrast, we only observed an impairment in fear extinction in WLH comorbid with PTSD in this study.

When we looked at the relationship between PTSD symptom severity and fear extinction to assess the continuous impacts of total PTSD symptoms, as opposed to categorical impact of a probable PTSD diagnosis, we found a negative correlation that was only observed in WLH. This result suggests that the fear extinction impairment in WLH due to PTSD is dimensional in nature, such that greater PTSD symptoms are associated with fear extinction deficits regardless of if someone meets diagnostic criteria for PTSD.

The absence of an effect on fear extinction in women with only PTSD could be due to the limited number of participants with probable PTSD. The prevalence of probable PTSD in this study is reflective of the prevalence both within the general population and within the population of WLH,^9,40^ but the low representation in these groups increases the risk of Type II error. Alternatively, the observation of impaired extinction for WLH and comorbid PTSD suggests that HIV status modulates the impact of PTSD on fear inhibition during extinction, a difference that cannot be attributed to income and education as these factors were corrected for in these analyses. A potential explanation for this modulating role of HIV status is the reported influence of HIV on cognitive processes leading to deficits in learning.^41^

In the context of HIV, an association between stress and deficits in verbal learning and memory has been demonstrated and further linked to decreased brain volume in specific regions associated with learning and memory.^42^ In addition, other studies have shown significant neurocognitive deficits in WLH with high trauma exposure,^43,44^ similar to the exposure of participants in our study. Our results and the extant literature suggest that altered fear neurocircuitry function or structure may underly the impaired fear extinction in WLH comorbid with PTSD observed in this study.

Although preclinical models of fear conditioning and extinction^45–47^ and human functional magnetic resonance imaging (fMRI) studies^48–51^ have resulted in a well-defined neurocircuitry for fear learning and extinction, the impact of HIV on this neurocircuitry remains unclear. The key regions of the fear neurocircuitry include the amygdala, ventromedial prefrontal cortex (vmPFC), and hippocampus. As noted above, there has been a report of enlarged amygdala volumes in PLWH.^34^

Subregions within the amygdala have special roles in mediating fear acquisition and extinction. Basal amygdala neurons synapse onto intercalated neurons, which are thought to be responsible for the fear inhibition that occurs during fear extinction.^52,53^ Intercalated neurons synapse onto central nucleus of the amygdala neurons, a region responsible for both acquisition and expression of fear.^54,55^ Our finding that HIV interacts with PTSD to impact fear extinction, but not fear acquisition, suggests potential differential effects of HIV on these intercalated neurons and their function in the basal versus central nucleus of the amygdala.

Although the amygdala is largely responsible for fear acquisition, it is also involved in fear extinction along with the vmPFC.^56^ The vmPFC downregulates the activity of the amygdala by activating the intercalated neurons when fear responses are not appropriate, such as during extinction. Greater amygdala reactivity, lower vmPFC activation, and poorer functional connections between the amygdala and the vmPFC have been observed in individuals with PTSD, suggesting impaired inhibition of the amygdala by the vmPFC.^57–59^

The hippocampus is central to contextual learning and memory and provides contextual information used for inhibition of the fear response. Smaller hippocampal volumes have consistently been observed in individuals with PTSD,^60,61^ and altered hippocampal functioning is associated with PTSD development and symptom severity.^62,63^ Similarly, fMRI studies of PLWH have provided evidence of decreased hippocampal activation during periods of encoding and retrieval, suggesting an association between hippocampal dysfunction and HIV,^64,65^ but functional assessments of the amygdala and neural circuitry during fear acquisition and extinction have not yet been reported for men or WLH.

In conclusion, this study shows that fear extinction is most severely impaired in individuals with HIV and comorbid PTSD. Because fear extinction is the primary mechanism on which trauma-focused therapy for PTSD is based,^39,66^ these results suggest that it may be important for care providers to consider HIV status when treating PTSD as the recommended exposure-based therapies may be less effective. The efficacy of trauma-focused PTSD treatments in PLWH is an evolving area of research that requires more exploration to uncover the mechanisms underlying the enhanced fear response deficits in this group of at-risk individuals.

Follow-up studies with greater representation of women and men with PTSD are necessary and should incorporate metrics of early life trauma as well as better control for types of antiretroviral therapies used by participants. Future studies may also incorporate clinician-administered assessments with formal PTSD diagnosis, as this study was limited by use of a self-report PTSD measure. Finally, it will be critical to extend the reported findings to examine the mechanisms underlying fear learning deficits. Future work may use fMRI to examine specific brain regions within the fear circuitry to better understand mechanisms of HIV impacting fear extinction, which will be needed to develop trauma-focused treatments tailored to WLH.

## Abbreviations Used

ANCOVA: analyses of covariance
ANOVA: analysis of variance test
ART: antiretroviral therapy
CSs: conditioned stimuli
CS+: reinforced conditioned stimulus
Cs–: non-reinforced conditioned stimulus
EMG: Electromyographic
fMRI: functional magnetic resonance imaging
FPS: fear-potentiated startle
PLWH: people living with HIV
PTSD: posttraumatic stress disorder
TEI: traumatic events inventory
USs: unconditioned stimuli
VmPFC: ventromedial prefrontal cortex
WLH: women living with HIV
